# Supplementary material for: Prominent involvement of acetylcholine dynamics in stable olfactory representation across the Drosophila brain
Source: Nat Commun. 2025 Sep 30;16:8638. doi: 10.1038/s41467-025-63823-2 (PMC12485117; doi:10.1038/s41467-025-63823-2)
Supplement: Supplementary file 1 — Supplementation Information [file 41467_2025_63823_MOESM1_ESM.pdf]

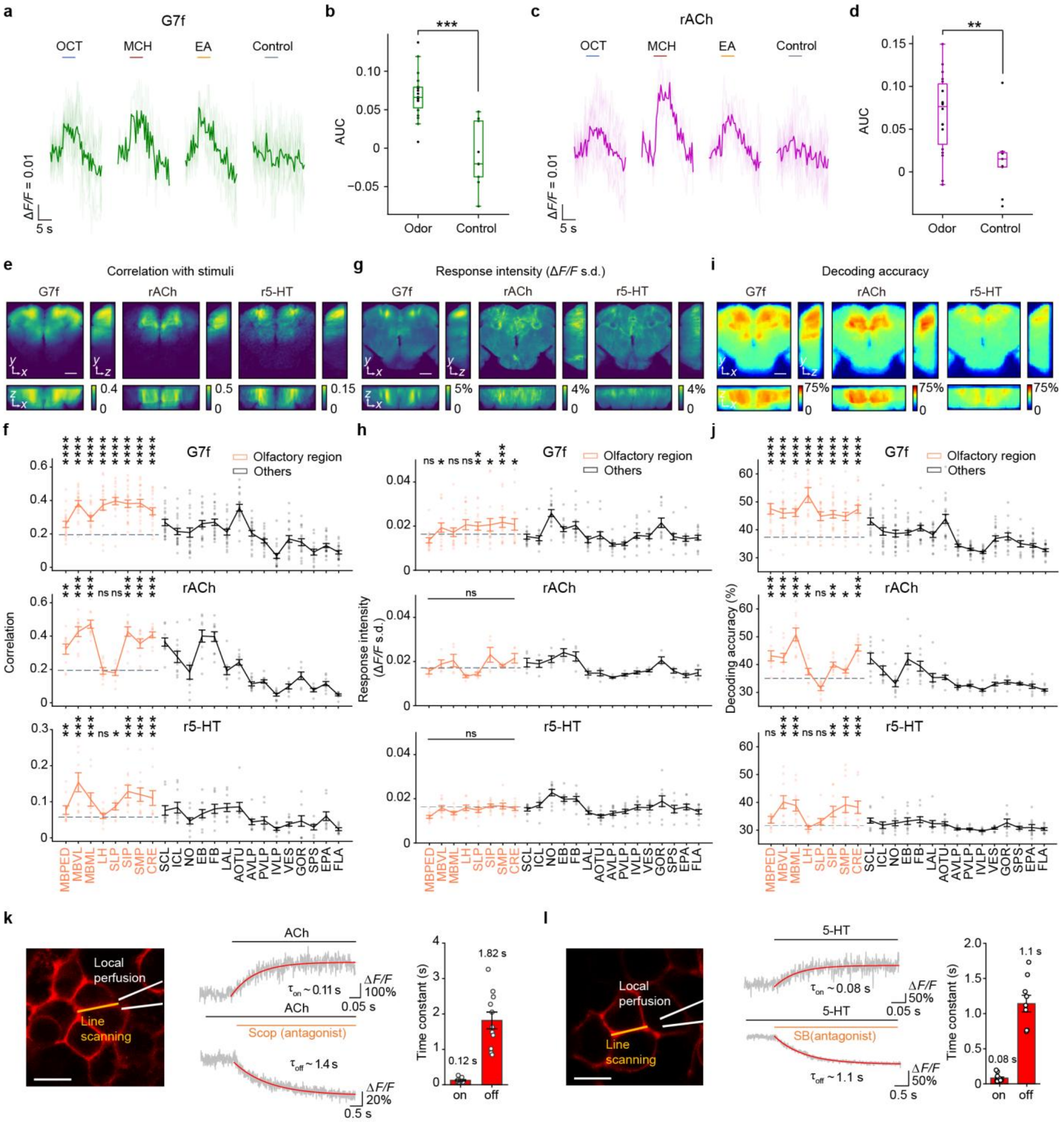

Supplementary Fig. 1.

Olfactory response across brain regions and indicator kinetics. a-d, Control experiments assessing neural responses to the brief air-puffs. a, *ΔF/F* of G7f in response to OCT, MCH, EA, and the brief air-puff (control) in a region of interest (ROI) within the olfactory regions from the center view obtained by 2pSAM. Dark: trial average; light: individual trials (3 trials of OCT, 1 trial of MCH, 2 trials of EA, and 3 trials of control in a pseudo-random order for each fly; 3flies

with the genotype w; UAS-rGRAB_ACh-0.5/+; nSyb-Gal4, UAS-jGCaMP7f). b, Box plots of the area under the curve (AUC) during odor stimulus periods for odors and control (n = 18 for Odor, n = 9 for Control). The dots represent the AUC values of each trial. One-sided Mann-Whitney U test applied. c, d, Similar to a, b, but for rACh responses. e-j, Measurements of responsiveness, response intensity, and odor identity classification accuracy across brain regions. e, Maps of correlation between G7f, rACh, and r5-HT dynamics and odor stimuli (averaged across flies, n = 20 for G7f, n = 10 for rACh, n = 10 for r5-HT). f, The average of the top 20% correlation values for each region (mean ± s.e.m). g, Maps of the average standard deviation of $\Delta F/F$ during odor stimulation across trials for G7f, rACh, and r5-HT (averaged across flies, n = 15 for G7f, n = 5 for rACh, n = 10 for r5-HT). h, The average standard deviation of each region (mean ± s.e.m). i, Maps of odor identity classification accuracy for G7f, rACh, and r5-HT (averaged across flies, n = 20 for G7f, n = 10 for rACh, n = 10 for r5-HT). j, The average odor identity classification accuracy of each region (mean ± s.e.m). The gray dashed lines indicate the average level of the non-olfactory regions in f, h, j. One-sided Wilcoxon signed-rank test for each olfactory brain region against the average of the non-olfactory regions performed in f, h, j. k, l, Assessment of the kinetics of rACh and r5-HT. k, Kinetics of rACh expressed in HEK293T cells. Left, Schematic of the local puffing system. Middle, Representative traces of rACh fluorescence increase in response to ACh (top) and decrease in response to Scop (bottom). Right, Group summary of on and off kinetics for rACh. n=11 cells for on and off kinetics, mean ± s.e.m. l, Kinetic analysis of r5-HT, similar to k. Scale bars: 50 μm in e, g, i, 20 μm in k, l. Source data are provided as a Source Data file. ****$P < 0.0001$, ***$P < 0.001$, **$P < 0.01$, *$P < 0.05$, ns - not significant ($P > 0.05$).

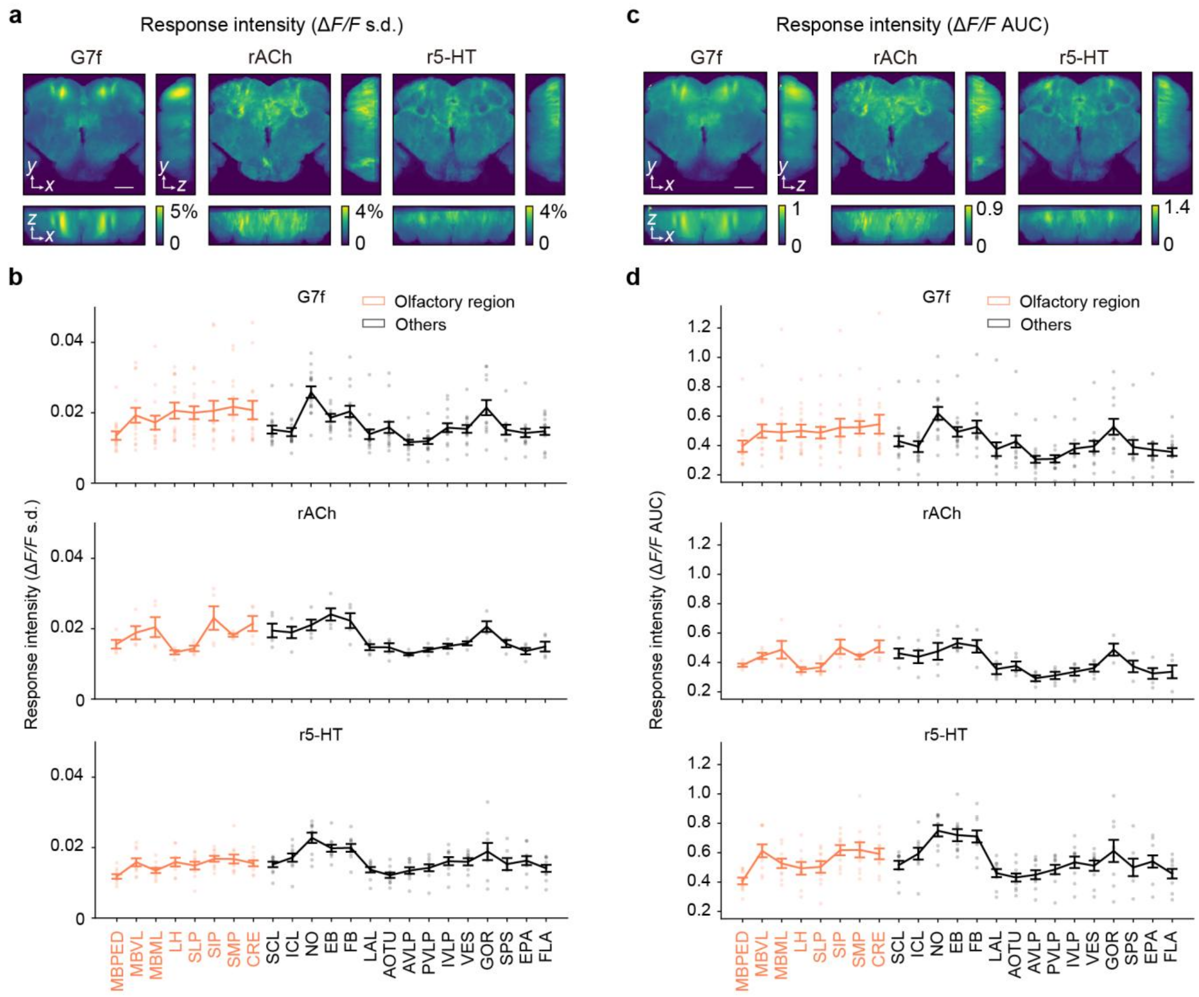

Supplementary Fig. 2.

Response intensity across the brain. a, Maps of the average standard deviation of Δ*F/F* during odor stimulation across trials for G7f, rACh, and r5-HT (averaged across flies, n = 15 for G7f, n = 5 for rACh, n = 10 for r5-HT). b, The average standard deviation of each region (mean ± s.e.m, n = 15 for G7f, n = 5 for rACh, n = 10 for r5-HT). c, d, Similar to a, b, but for AUC. 10 flies co-labeled by G7f and rACh and 10 flies co-labeled by G7f and r5-HT are analyzed. Flies co-labeled by G7f and rACh with high-intensity non-specific fluorescence on the upper edge of the brain are excluded for clarity. Scale bars: 50 μm in a, c.

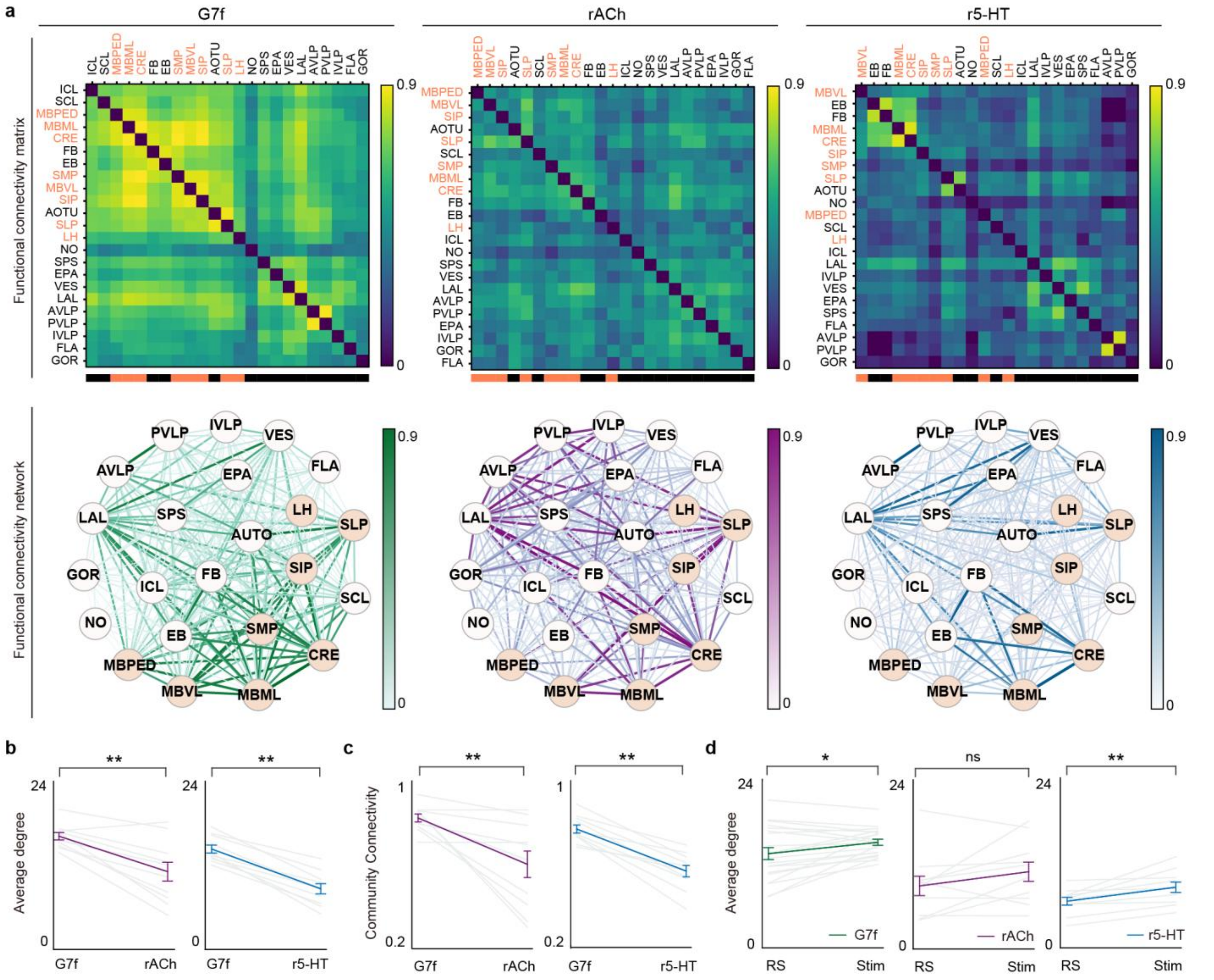

Supplementary Fig. 3.

The functional connectivity networks of three signals during different states show distinct properties. a, Functional connectivity matrices and networks of the left-side brain regions and the central complex for each signal in the resting state. The order of the matrix elements and the placement of the network nodes are consistent with Fig. 2e. b, c, The average degree and community connections of the functional connectivity networks during odor stimulation for different co-labeled indicators. d, The average degree of the functional connectivity networks in different states. In b-d, each light-colored line represents a fly.10 flies co-labeled by G7f and rACh and 10 flies co-labeled by G7f and r5-HT are analyzed. *n* = 20 flies for G7f, *n* = 10 flies for rACh, *n* = 10 flies for r5-HT, mean ± s.e.m in a and d. *n* = 10 flies, mean ± s.e.m in b, c. Stim: Odor stimulation. RS: The resting state. Two-sided Wilcoxon signed-rank test. Source data are

provided as a Source Data file. ****$P < 0.0001$, ***$P < 0.001$, **$P < 0.01$, *$P < 0.05$, ns - not significant ($P > 0.05$).

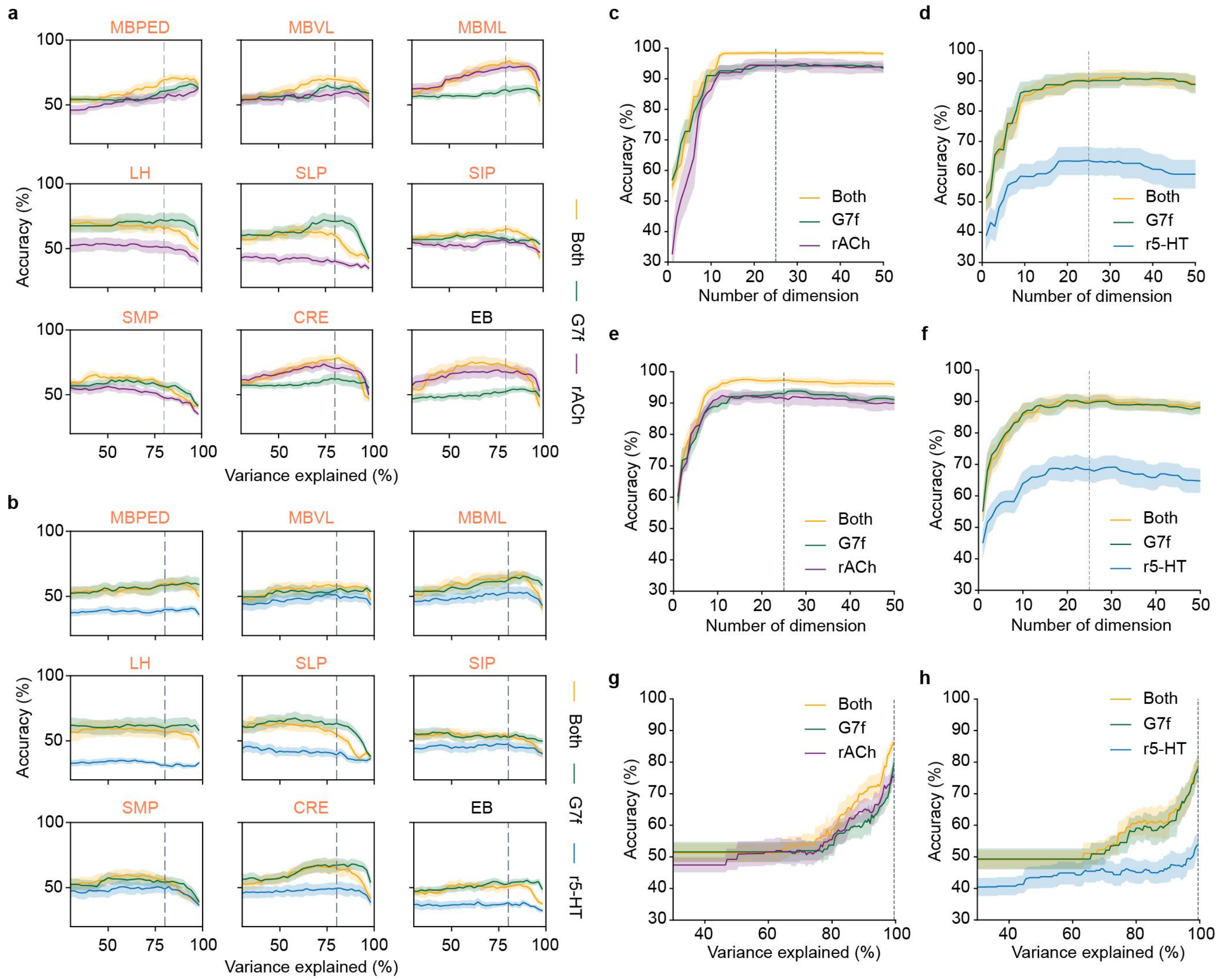

Supplementary Fig. 4.

PCA threshold determination for odor identity classifications. a, The accuracy of the voxel-level odor identity classification in 9 brain regions (left-side olfactory brain regions and EB) changing with the explained variance of the PCs retained, for G7f, rACh, and integrating both channels. The accuracy does not show significant change with the explained variance. A threshold of 0.8 is taken as most brain regions reach a high and stable level of accuracy (see Methods), marked by the dashed line. b, Similar to a, but for flies co-labeled by G7f and r5-HT. c, The accuracy of the voxel-level multiple-brain-region odor identity classification changing with the dimensions of PCs retained (Step 3 in Fig. 3c), for G7f, rACh, and integrating both

channels. Integrating both channels yields a higher accuracy for almost all dimension values. The dashed line (dimension = 25) marks the threshold taken, as the average accuracy reaches a high and stable level for all indicators (see Methods). d, Similar to c, but for flies co-labeled by G7f and r5-HT. c, d, are results using the denoising algorithm SRDTrans. e, f, Similar to c, d, but are results using the denoising algorithm DeepCAD-RT. Obvious accuracy gains for integrating rACh and G7f signals exist using both denoising algorithms. g, The accuracy of the region-level multiple-brain-region odor identity classification changing with the explained variance of the PCs retained, for G7f, rACh, and integrating both channels. A threshold of 0.995 is taken, marked by the dashed line (see Methods). h, Similar to g, but for flies co-labeled by G7f and r5-HT. 10 flies co-labeled by G7f and rACh and 10 flies co-labeled by G7f and r5-HT are analyzed. n = 10, mean ± s.e.m (shades). Flies without a general coverage of region LH are excluded from the statistics of this region. Source data are provided as a Source Data file.

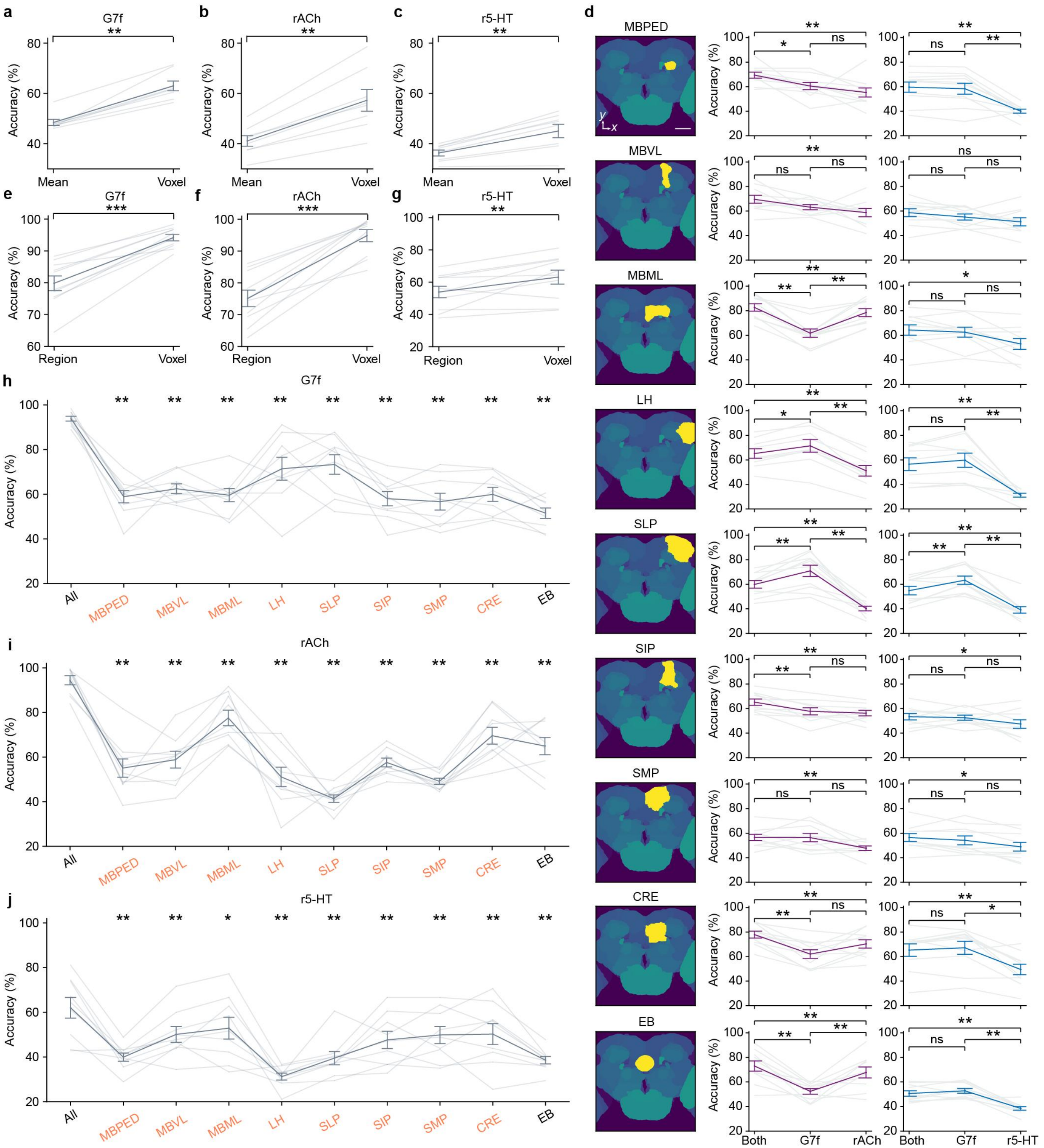

Supplementary Fig. 5.

Comparison of odor identity classification accuracies across channels and scales. a-c, Statistics of the average odor classification accuracy of the blocks and the accuracy integrating all voxels in each olfactory region of the left semi-brain for G7f (a), rACh (b), and r5-HT (c). Each light-colored line represents the average accuracies of a region across all flies. n = 8 regions, mean ± s.e.m. d, Comparisons of the voxel-level odor identity classification accuracies

by different channels in each brain region (left-side olfactory brain regions and EB). Each region is highlighted in the atlas schematic diagram. e-g, Statistics of the odor identity classification accuracies of region-level and voxel-level data across multiple brain regions for G7f (e), rACh (f), and r5-HT (g). h-j, Statistics of the voxel-level odor identity classification accuracies across multiple brain regions and in single brain regions (left-side olfactory brain regions and EB) for G7f (h), rACh (i), and r5-HT (j). Hypothesis testing is performed between All and each region. 10 flies co-labeled by G7f and rACh and 10 flies co-labeled by G7f and r5-HT are analyzed. $n = 10$, mean ± s.e.m in d-j. Results of the G7f channel of the flies co-labeled by G7f and rACh are shown in a, e, and h. Flies without a general coverage of region LH are excluded from the statistics of this region in a-d. Flies without a general coverage of region LH are excluded for clarity in h-j. Each light-colored line in the statistical graphs represents the accuracies of a fly in d-j. One-sided Wilcoxon signed-rank test in a-c, e-j. Two-sided Wilcoxon signed-rank test in d. Benjamini/Hochberg multi-comparison correction applied in d. Source data are provided as a Source Data file. ****$P < 0.0001$, ***$P < 0.001$, **$P < 0.01$, *$P < 0.05$, ns - not significant ($P > 0.05$). Scale bar: 50 μm in d.

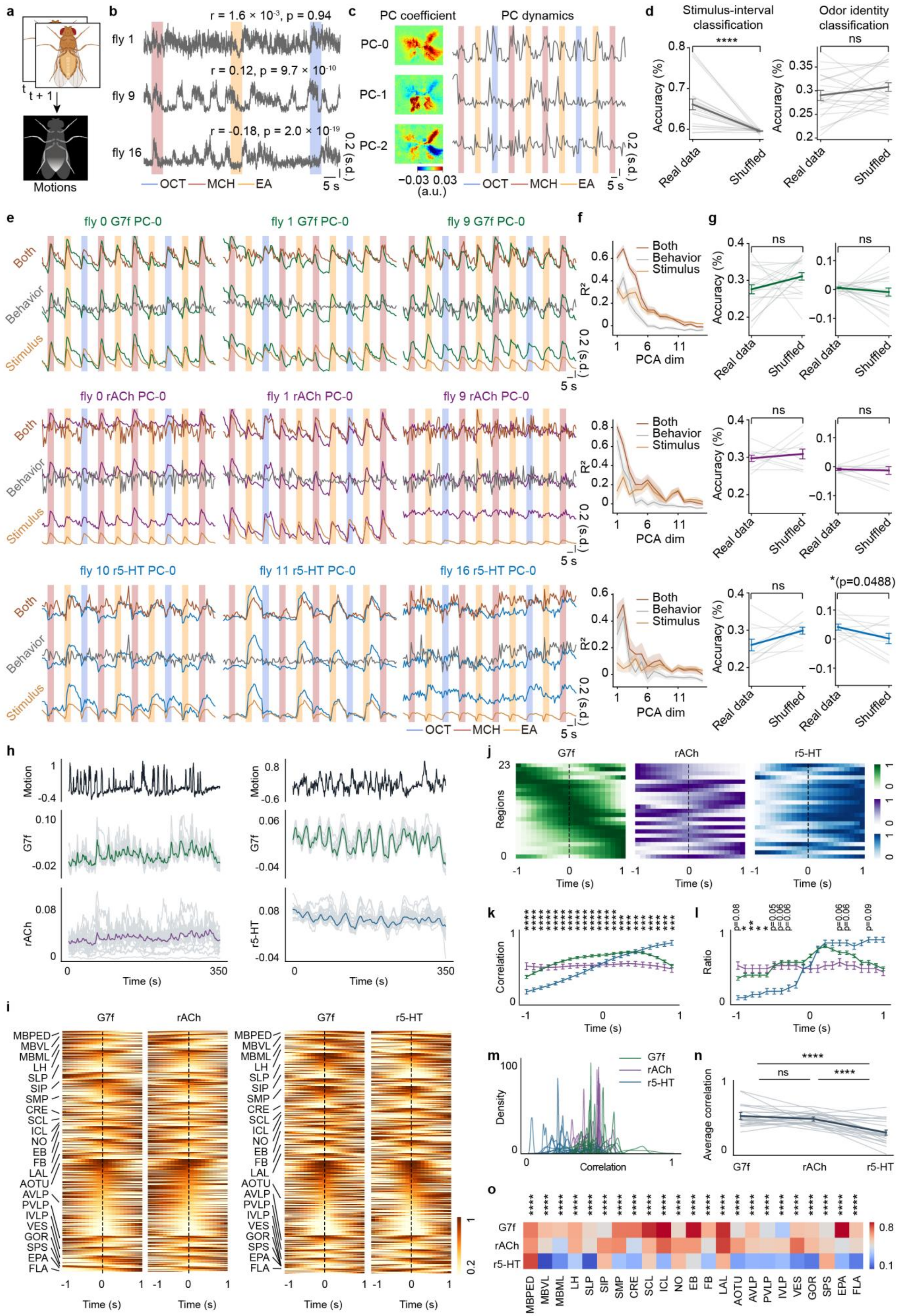
a
t
t + 1
Motions
b
r = 1.6 × 10⁻³, p = 0.94
fly 1
r = 0.12, p = 9.7 × 10⁻¹⁰
fly 9
r = -0.18, p = 2.0 × 10⁻¹⁹
fly 16
0.2 (s.d.)
5 s
OCT
MCH
EA
c
PC coefficient
PC dynamics
PC-0
PC-1
PC-2
-0.03 0.03
(a.u.)
d
Stimulus-interval classification
Odor identity classification
****
ns
Accuracy (%)
Real data
Shuffled
e
fly 0 G7f PC-0
fly 1 G7f PC-0
fly 9 G7f PC-0
Both
Behavior
Stimulus
fly 0 rACh PC-0
fly 1 rACh PC-0
fly 9 rACh PC-0
fly 10 r5-HT PC-0
fly 11 r5-HT PC-0
fly 16 r5-HT PC-0
f
R²
PCA dim
g
*(p=0.0488)
h
Motion
G7f
rACh
r5-HT
Time (s)
i
MBPED
MBVL
MBML
LH
SLP
SIP
SMP
CRE
SCL
ICL
NO
EB
FB
LAL
AOTU
AVLP
PVLP
IVLP
VES
GOR
SPS
EPA
FLA
j
Regions
k
Correlation
l
Ratio
m
Density
Correlation
n
Average correlation
o

Supplementary Fig. 6.

Motion modulates neuronal activities and neurochemical dynamics but does not account for odor identity representation. a, Motions are extracted by subtracting consecutive frames of the videos recording fly abdomens and computing the absolute differences. Created in BioRender. Fan, J. (2025) https://BioRender.com/cp0dmbd. b, Dynamically changing motion energy of the flies. The correlation between odor stimuli and motion energy is diverse and can be weak (top), positive (middle), or negative (bottom). c, PCA is performed on the videos to reduce the dimensionality and extract the behavioral features. Left: PC coefficients mapped on the FOV. Right: The dynamics of the PCs in a short period. d, Left: The classification accuracy of stimulus periods and intervals. Right: The classification accuracy of odor identities. Shuffled refers to shuffling the labels. e, Regression of neuronal activity principle components by stimulus (gold, bottom), behavior (gray, middle), and both (brown, top). f, R^2 of the regression. The results of the regression of the first 15 PCs are shown. g, Left: The classification accuracy of odor identities by the partial neuronal PCs explained by behavior. Right: The change of the classification accuracy of odor identities by removing the neuronal components explained by behavior. Shuffled refers to shuffling the labels. The shades sign odor stimulus periods (blue: OCT; red: MCH; orange: EA) in b, c, and e. h, Motion and neural dynamics during the same time period. i, Correlation matrices between motion and neural dynamics of voxel levels across multiple brain regions. The horizontal coordinate indicates the time lag of the neural activity in terms of the zero point of the motion, where the positive values represent neural dynamics delayed from motion and negative values represent neural dynamics advanced from motion. The horizontal coordinates of j, k and l have the same meaning. j, Correlation matrix between motion and neural dynamics at the brain region level. k, The average correlation for each time-delay condition. l, The proportion of correlations above the mean for each time-delay condition. m, The probability distributions of correlations for each signal. n, The average correlation for each signal. o, The average correlation in the 23 brain regions. Normalized correlations are used in i-o for presentation and calculation. 10 flies co-labeled by G7f and rACh and 10 flies co-labeled by G7f and r5-HT are analyzed. n = 20 flies for G7f and motion analyses, n = 10 flies for rACh, n = 10 flies for r5-HT, mean ± s.e.m. Two-sided Wilcoxon signed-rank test in d, g and n; two-sided Kruskal-Wallis test in k, l and o. Benjamini/Hochberg multi-comparison correction applied in n. Source data are provided as a Source Data file. ****$P < 0.0001$, ***$P < 0.001$, **$P < 0.01$, *$P < 0.05$, ns - not significant ($P > 0.05$, not shown in l).

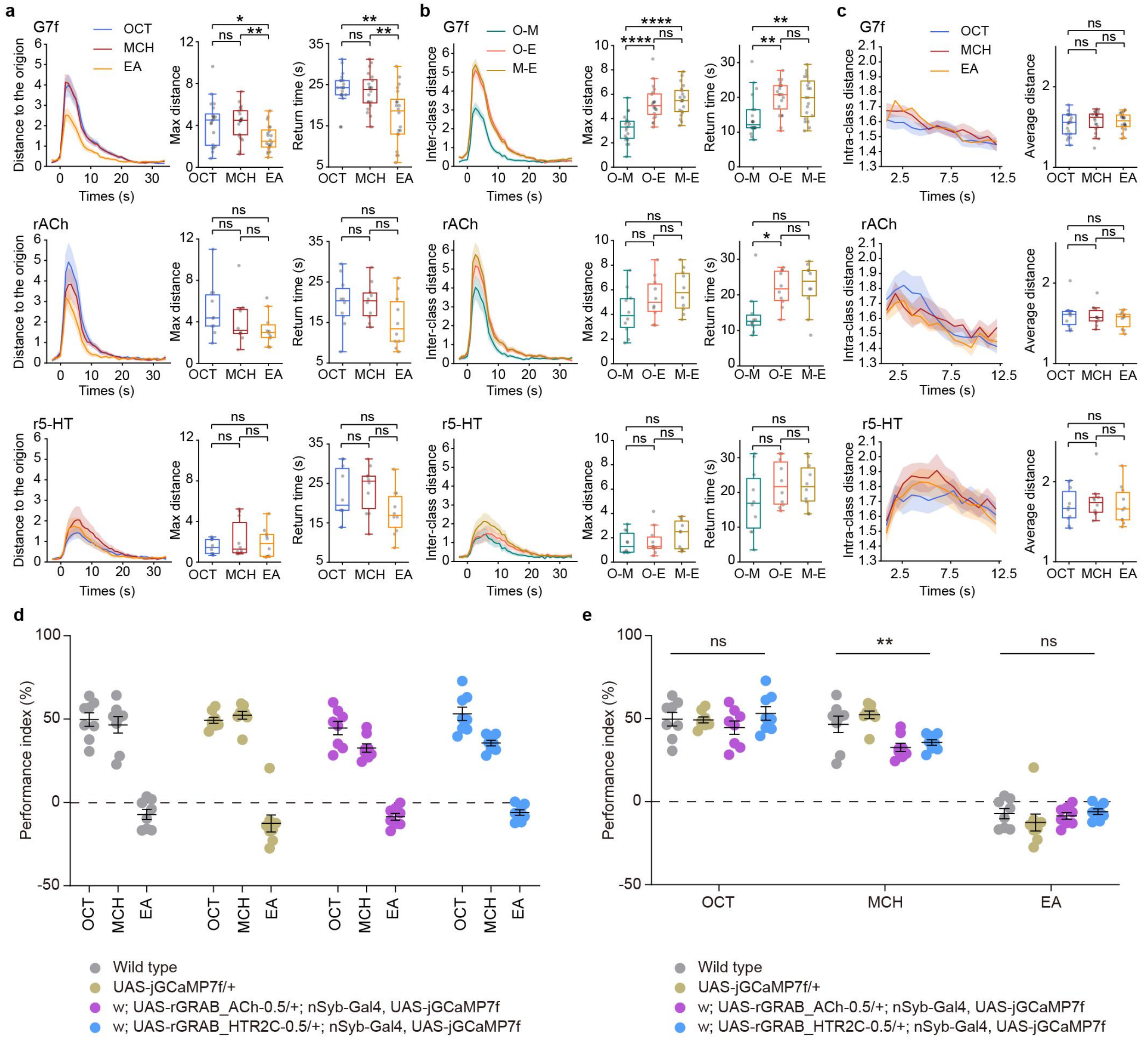

Supplementary Fig. 7.

Comparison of the manifolds of different odors. a, Comparison of the distance to the origin of different odors. Left: Average distance to the origin changing with time relative to odor delivery. Middle: Statistics of the maximum distance. Right: Statistics of the time for returning to the random state. The three rows show results of G7f, rACh, and r5-HT, from top to bottom, respectively. b, Similar to a, but for inter-class distance. O-M: OCT and MCH, O-E: OCT and EA, M-E: MCH and EA. c, Comparison of the intra-class distance of different odors. Left: Average intra-class distance changing with time relative to odor delivery. Right: Statistics of the average intra-class distance within the period shown in the left figure. 10 flies co-labeled by G7f

and rACh and 10 flies co-labeled by G7f and r5-HT are analyzed (n = 20 flies for G7f, n = 10 flies for rACh, n = 10 flies for r5-HT). Box plots: center line, median; box limits, upper and lower quartiles; whiskers, 1.5x interquartile range. Each point represents the result of a fly. Two-sided Mann-Whitney U test and Benjamini/Hochberg multi-comparison correction performed in a-c. d, e, Performance indices of flies with four genotypes in response to given odors (OCT / MCH / EA). 100 flies used in an experiment, n = 8 experiments, mean ± s.e.m. Kruskal-Wallis test in e. Source data are provided as a Source Data file. ****$P < 0.0001$, ***$P < 0.001$, **$P < 0.01$, *$P < 0.05$, ns - not significant ($P > 0.05$).

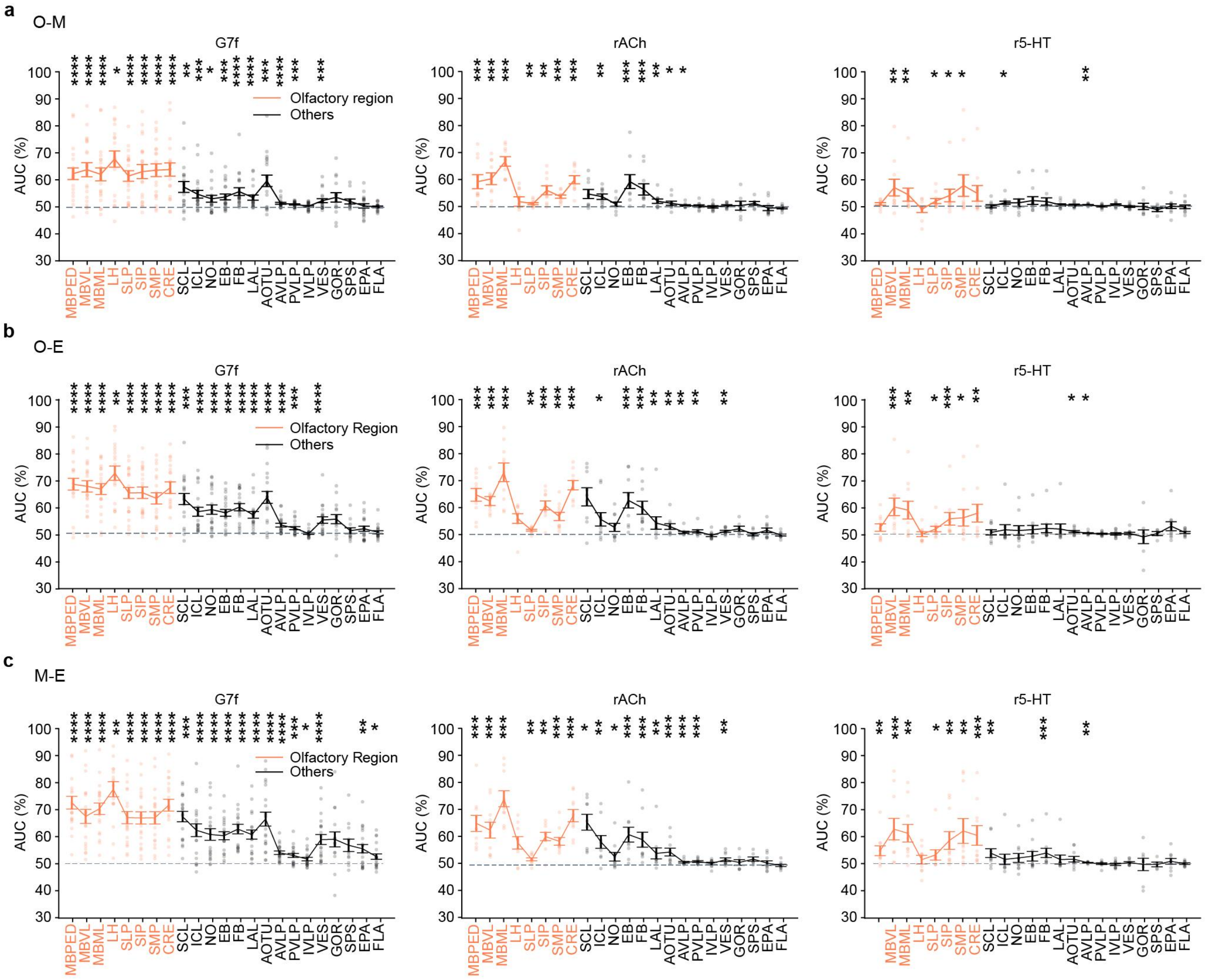

Supplementary Fig. 8.

Average 2-class classification AUC of the small blocks within each brain region for each odor pair. a-c, Average 2-class classification AUC between OCT and MCH (a), OCT and EA (b), MCH and EA (c) of each region for each indicator. $n = 20$ for G7f , $n = 10$ for rACh, $n = 10$ for r5-HT, mean ± s.e.m. 10 flies co-labeled by G7f and rACh and 10 flies co-labeled by G7f and r5-HT are analyzed. Gray lines represent the chance level of 2-class classification AUC (50%). One-sided Wilcoxon signed-rank test for each brain region against the chance level. ****$P < 0.0001$, ***$P < 0.001$, **$P < 0.01$, *$P < 0.05$, ns - not significant ($P > 0.05$, not labeled). O-M: OCT and MCH, O-E: OCT and EA, M-E: MCH and EA.

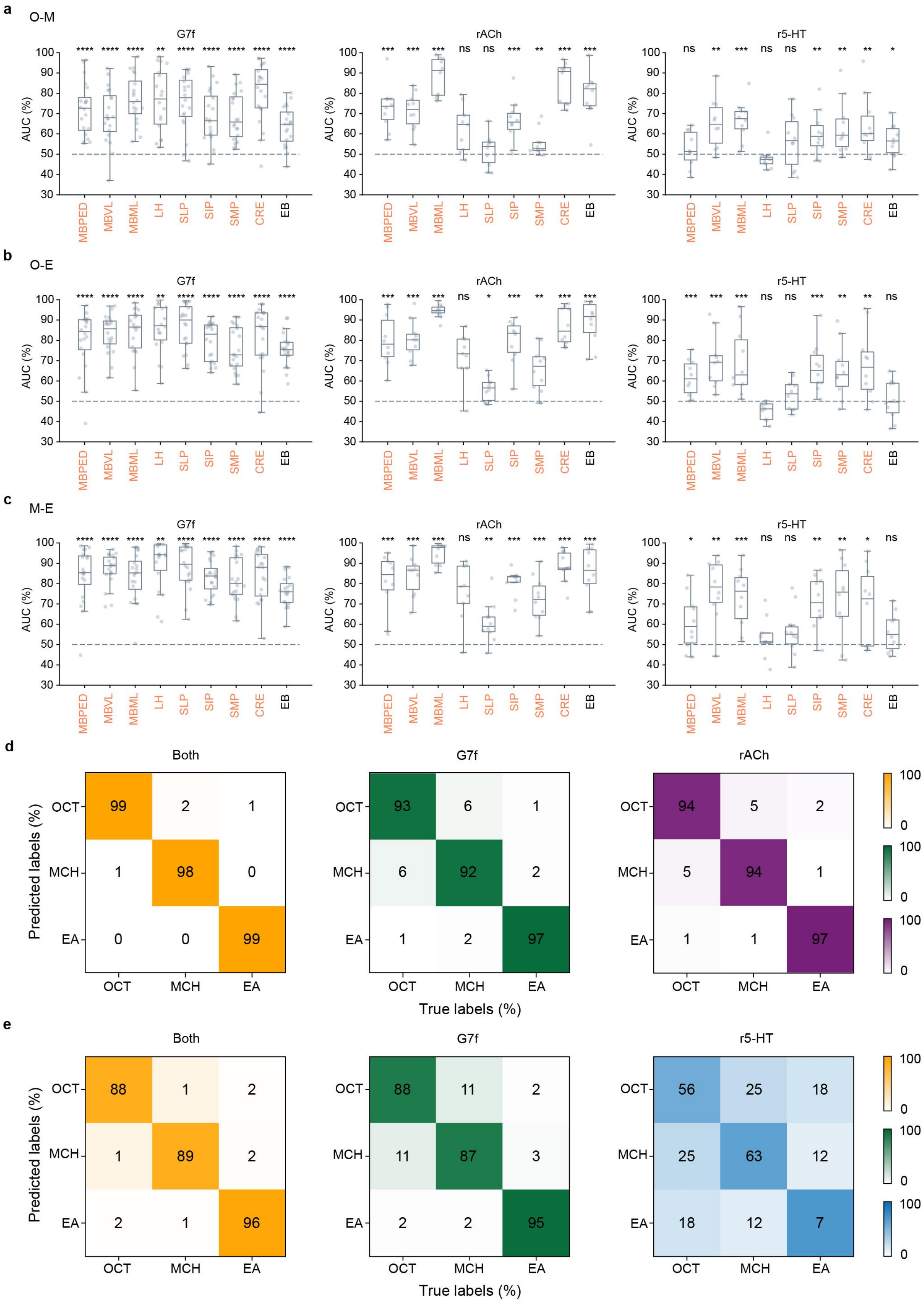
a
O-M
b
O-E
c
M-E
G7f
rACh
r5-HT
AUC (%)
MBPED
MBVL
MBML
LH
SLP
SIP
SMP
CRE
EB
d
Both
G7f
rACh
Predicted labels (%)
True labels (%)
OCT
MCH
EA
99
2
1
1
98
0
0
0
99
93
6
1
6
92
2
1
2
97
94
5
2
5
94
1
1
1
97
e
Both
G7f
r5-HT
88
1
2
1
89
2
2
1
96
88
11
2
11
87
3
2
2
95
56
25
18
25
63
12
18
12
7

Supplementary Fig. 9.

2-**class classification accuracy of each brain region and across multiple brain regions for each odor pair.** a-c, 2-class classification AUC between OCT and MCH (a), OCT and EA (b), MCH and EA (c) of each region for each indicator. *n* = 20 for G7f , *n* = 10 for rACh, *n* = 10 for r5-HT. Box plots: center line, median; box limits, upper and lower quartiles; whiskers, 1.5x interquartile range. Each point represents the result of a fly. 10 flies co-labeled by G7f and rACh and 10 flies co-labeled by G7f and r5-HT are analyzed. Gray lines represent the chance level of 2-class classification AUC (50%). One-sided Wilcoxon signed-rank test for each brain region against the chance level. ****$P < 0.0001$, ***$P < 0.001$, **$P < 0.01$, *$P < 0.05$, ns - not significant ($P > 0.05$). O-M: OCT and MCH, O-E: OCT and EA, M-E: MCH and EA. d, Confusion matrices of odor identity classification for integrating G7f and rACh, G7f, and rACh (*n* = 10, averaged across flies). Color and values denote the ratio of the predicted and true class. e, Confusion matrices of odor identity classification for integrating G7f and r5-HT, G7f, and r5-HT (*n* = 10, averaged across flies), similar to d.

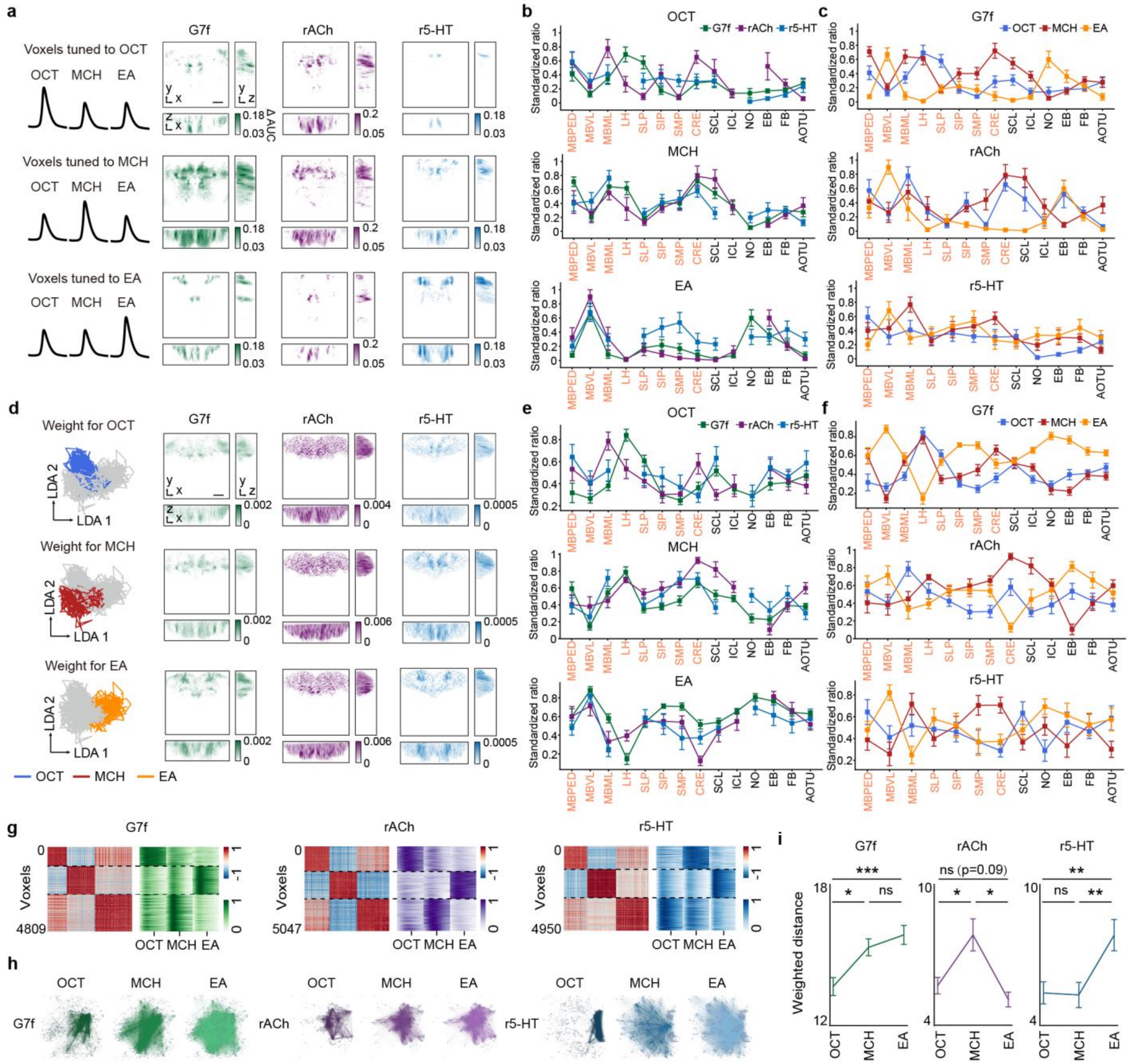

Supplementary Fig. 10.

Ensembles of odor representations. a, Maps of odor tuning. Colors denote the average difference of AUC of the responses to the specific odor compared to the other two odors (averaged across flies, n = 20 for G7f, n = 10 for rACh, n = 10 for r5-HT). b, c, Statistics of the standardized ratio of voxels with specific tuning in each region (mean ± s.e.m, n = 20 for G7f, n = 10 for rACh, n = 10 for r5-HT). Regions within the community containing most olfactory regions for each indicator are displayed. Each chart shows the result for a specific tuning in b, results for a specific indicator in c. d-f, Similar to a-c, but for odor identification weight. g, Functional connectivity matrix and traces of voxel ensembles. The connectivity matrix is

clustered into 3 modules by hierarchical clustering methods. h, Functional connectivity network of three odors. The nodes are the ensemble of voxels that responded strongly to odor stimuli, and the connectivity edges shown are the top 10% functional edges with the highest correlations. i, Comparison of weighted distances (mean ± s.e.m, n = 20 for G7f, n = 10 for rACh, n = 10 for r5-HT). The weighted distance was calculated as the physical spatial distance between a pair of voxels multiplied by the functional correlation between them. Two-sided Wilcoxon signed-rank test and Benjamini/Hochberg multi-comparison correction performed. 10 flies co-labeled by G7f and rACh and 10 flies co-labeled by G7f and r5-HT are analyzed. Source data are provided as a Source Data file. ****$P < 0.0001$, ***$P < 0.001$, **$P < 0.01$, *$P < 0.05$, ns - not significant ($P >$ 0.05).

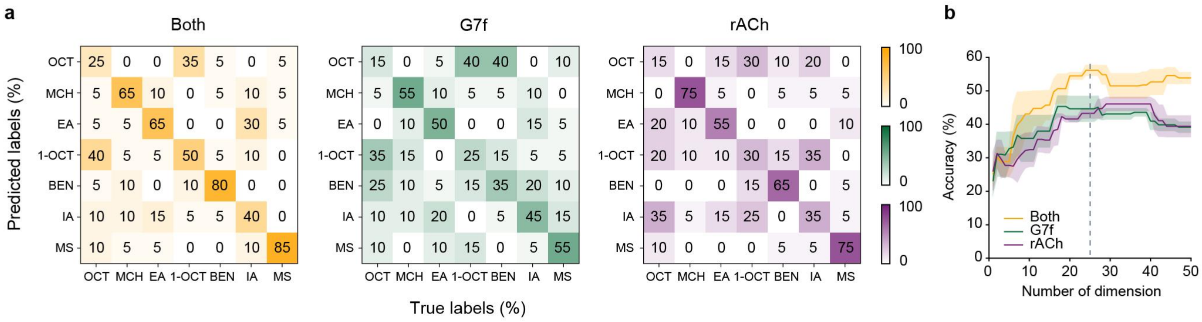

Supplementary Fig. 11.

Odor identity classification of 7 odors at the multiple-brain-region scale. a, Confusion matrices of odor identity classification for integrating G7f and rACh, G7f, and rACh of a fly. Color and values denote the ratio of the predicted and true class. b, The accuracy of the voxel-level multiple-brain-region odor identity classification changing with the dimensions of PCs retained (Step 3 in Fig. 3c), for G7f, rACh, and integrating both channels (n = 3, mean ± s.e.m). Integrating both channels yields a higher accuracy for almost all dimension values. The dashed line (dimension = 25) marks the threshold taken, as the average accuracy reaches a high and stable level for all indicators. OCT: 3-octanol, MCH: 4-methylcyclohexanol, EA: ethyl acetate, 1-OCT: 1-Octen-3-ol, BEN: Benzaldehyde, IA: Isopentyl acetate, MS: Methyl salicylate.

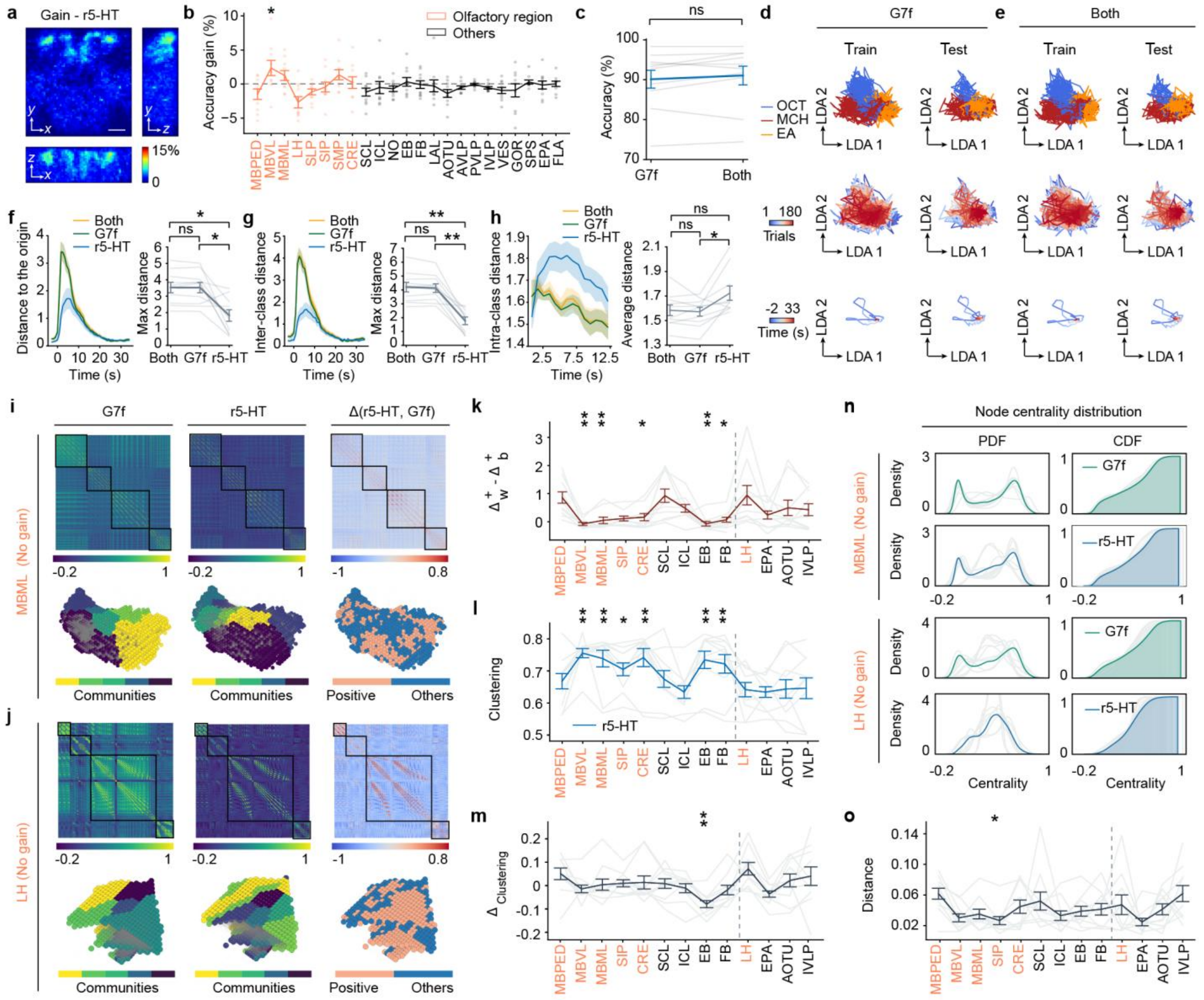

Supplementary Fig. 12.

Limited enhancement of odor identity representation by integrating 5-HT dynamics. a, Map of accuracy gain integrating r5-HT dynamics averaged across flies. b, Statistics of the average accuracy gain in each brain region. c, Comparison of the voxel-level multiple-brain-region odor identity classification accuracies between using only the G7f channel and integrating both channels. d, e, Low-dimensional manifolds by G7f (d) and both channels (e) of a fly, similar to Fig. 4d, e. The manifolds are from the same fly as Fig. 3f. Arrow lengths are in arbitrary units but consistent across dimensions. f-h, Metrics of the manifolds for integrating both channels, G7f and r5-HT. f, Distance to the origin. g, Inter-class distance. h, Intra-class distance. i, j, Functional connectivity of the voxels within two brain regions, the MBML (i) and the LH (j). The three columns show the functional connectivity of G7f, r5-HT, and their deflation

ratio difference, from left to right. For the MBML, connectivity emphasis exists without the presence of obvious connectivity complementation to G7f by r5-HT (top). Highly connected voxels do not show evident spatial clustering (bottom, coral). For the LH, similar results are shown. k, The difference in increased deflation ratios within and between clusters of the functional connectivity of r5-HT compared to G7f. l, The average clustering coefficient of each brain region for r5-HT. m, The average clustering coefficient difference between r5-HT and G7f in each region. n, The node centrality distribution of MBML and LH. o, The distance of the node centrality distributions between r5-HT and G7f in each region. In k, l, m, and o, hypothesis testing is performed as Fig. 4. $n = 10$ flies co-labeled by G7f and r5-HT, mean ± s.e.m. Results of the left-side brain regions and the central complex are shown in b, k-o. Each light-colored line represents the result of a fly in c, f-h, and k-o. One-sided Wilcoxon signed-rank test in b, c, k-m, o; Two-sided Wilcoxon signed-rank test in f-h; Benjamini/Hochberg multi-comparison correction applied in f-h. Source data are provided as a Source Data file. ****$P < 0.0001$, ***$P < 0.001$, **$P < 0.01$, *$P < 0.05$, ns - not significant ($P > 0.05$, not shown in b, k-m, o). Scale bar: 50 μm in a.

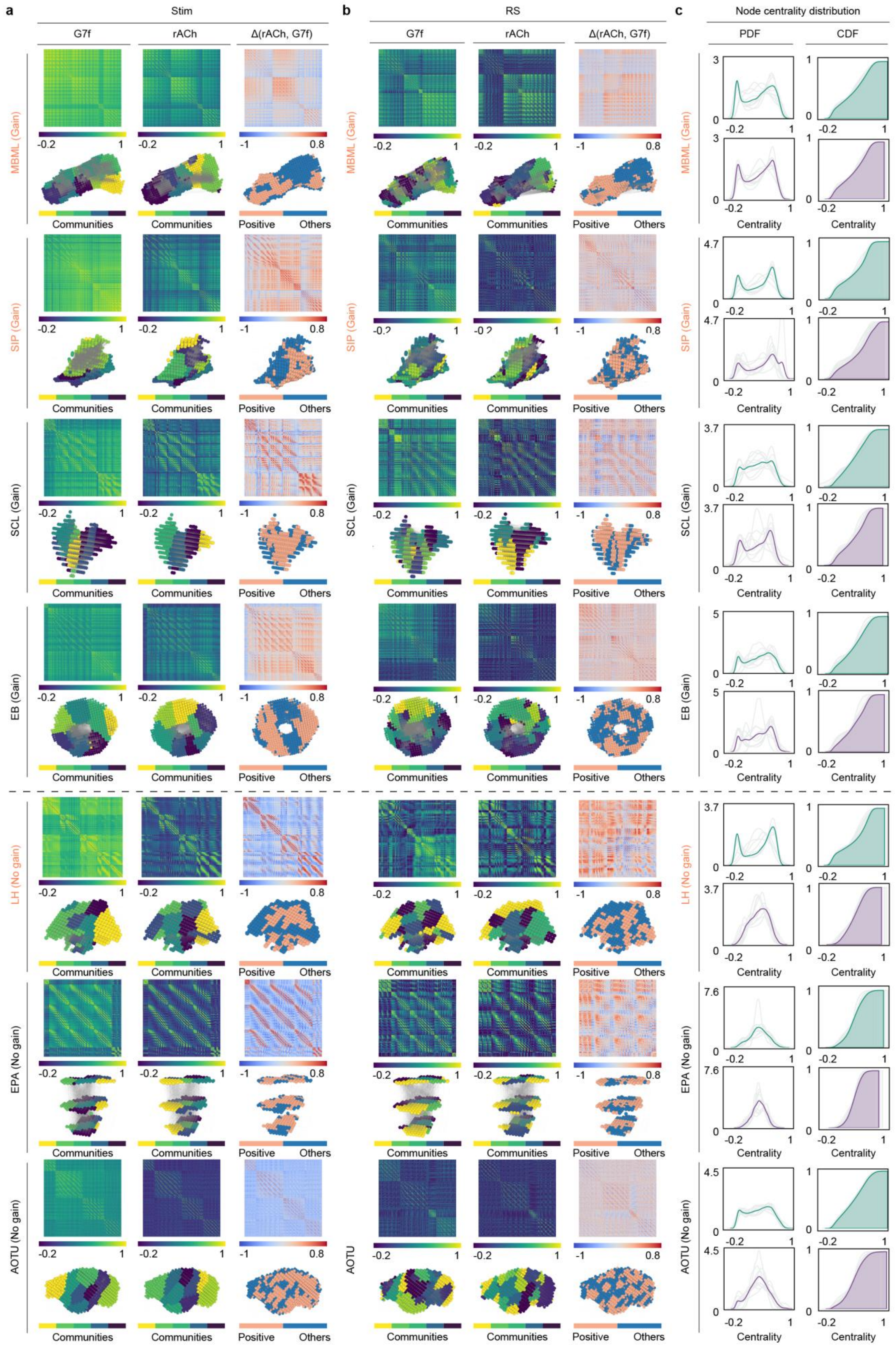
a
Stim
G7f
rAch
Δ(rACh, G7f)
b
RS
c
Node centrality distribution
PDF
CDF
MBML (Gain)
SIP (Gain)
SCL (Gain)
EB (Gain)
LH (No gain)
EPA (No gain)
AOTU (No gain)
AOTU
Communities
Positive
Others
Centrality

Supplementary Fig. 13.

Functional connectivity and topological characterizations by G7f and rACh in brain regions with and without accuracy gain. a, b, Functional connectivity matrices and networks by G7f and rACh in four brain regions with accuracy gain and three brain regions without accuracy gain during odor stimulation (a) and the resting state (b). These seven brain regions are selected from different neuropils of the *Drosophila* brain. For each brain region, three columns are descriptions of the functional connectivity of G7f, rACh, and the difference in the deflation ratio between them, from left to right (top). The functional connectivity of each brain region is shown according to the location of voxels in physical space (bottom). In a, for brain regions with accuracy gain, connectivity complementation and emphasis to G7f by rACh are apparent (top). The complementation is reflected in the different distribution of functional connections in the physical space (bottom). The community divisions are similar for the two channels (bottom, labeled in different colors), and voxels with the greatest increased connectivity gather together in space (bottom, coral). Connectivity emphasis exists for brain regions without accuracy gain without obvious complementation and gathering phenomenon. In b, the relationship between G7f and rACh is not clearly characterized in the brain regions either with or without accuracy gain. c, The node centrality distribution of the brain regions. Brain regions without accuracy gain tend to display more uniform functional connectivity for rACh. Stim: Odor stimulation. RS: The resting state. Each light-colored line represents the result of a fly in c.

254

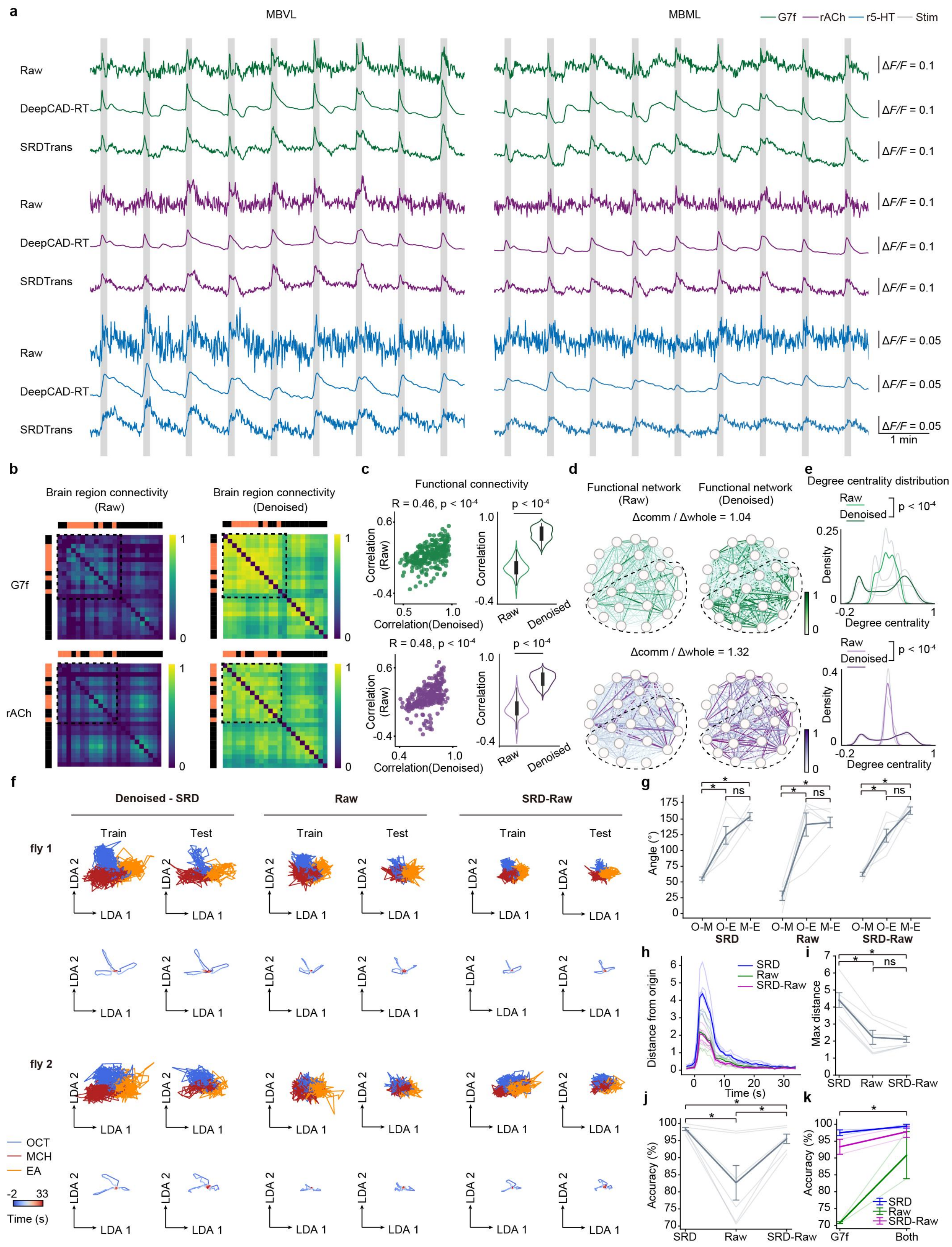

a
MBVL
MBML
G7f
rACh
r5-HT
Stim
Raw
DeepCAD-RT
SRDTrans
ΔF/F = 0.1
ΔF/F = 0.05
1 min
b
Brain region connectivity (Raw)
Brain region connectivity (Denoised)
G7f
rACh
c
Functional connectivity
R = 0.46, p < 10-4
R = 0.48, p < 10-4
Correlation (Raw)
Correlation(Denoised)
p < 10-4
Correlation
Raw
Denoised
d
Functional network (Raw)
Functional network (Denoised)
Δcomm / Δwhole = 1.04
Δcomm / Δwhole = 1.32
e
Degree centrality distribution
Density
Degree centrality
f
Denoised - SRD
Raw
SRD-Raw
Train
Test
fly 1
fly 2
LDA 1
LDA 2
OCT
MCH
EA
Time (s)
g
Angle (°)
O-M O-E M-E
SRD
Raw
SRD-Raw
ns
h
Distance from origin
Time (s)
i
Max distance
j
Accuracy (%)
k
G7f
Both

Supplementary Fig. 14.

Effects of the denoising algorithms. a, Δ*F/F* of two brain regions for three indicators, before and after denoising with DeepCAD-RT and SRDTrans. b,Functional connectivity matrices of brain regions during odor stimulation for G7f and rACh averaged across two flies, before and after denoising. The dashed boxes sign the communities containing most olfactory regions identified in Fig. 2. The color blocks above and to the left of each matrix mark 8 olfactory (coral) and 15 non-olfactory regions (black). c, Left: Scatter plots of the pairwise correlations between functional connectivity matrices for raw and denoised data. Pearson correlation is calculated, and R and *P* statistics are labeled. Right: Statistics of the correlation values. Kolmogorov-Smirnov test applied. d, Functional connectivity networks based on the matrices. The colors and widths of the edges indicate normalized connection strengths. The dashed circles sign the communities as in b. e, Node degree centrality distribution. Light-colored lines for raw data and dark-colored lines for Denoised data. Each light gray lines represents the result of the denoised or raw data from a single fly. Kolmogorov-Smirnov test applied. f, Low-dimensional manifolds for integrating both channels of the two example flies. Top and: Each line is the odor response of a trial. Bottom: Each line is the average odor response of an odor identity. Manifolds from 2s before odor delivery to 33s after are plotted. Top: Colors denote odor identities (blue: OCT; red: MCH; orange: EA). Bottom: Colors denote the time relative to odor delivery (blue: early; red: late). The arrow lines are arbitrary units but indicate an equivalent length in each dimension. Three columns present manifolds for denoised data with SRDTrans (SRD), raw data (Raw), and training on denoised data and applying the transformation to raw data (SRD-Raw). g, Statistics of the angles between the trial-averaged low-dimensional manifolds of two odors (n = 6, mean ± s.e.m). O-M: OCT and MCH, O-E: OCT and EA, M-E: MCH and EA. Each light-colored line represents the manifold of integrating both channels, G7f or rACh of a fly. Two-sided Wilcoxon signed-rank test and Benjamini/Hochberg multi-comparison correction are applied. h, Distance to the origin of the manifolds changing with time relative to odor delivery. n = 6. Dark-colored lines represent the average of each situation, and each light-colored line represent the result of integrating both channels, G7f or rACh of a fly. i, Comparison of the maximum distance to the origin for SRD, Raw, and SRD-Raw (n = 6, mean ± s.e.m). Each light-colored line represents the result of integrating both channels, G7f or rACh of a fly. Two-sided Wilcoxon signed-rank test and Benjamini/Hochberg multi-comparison correction are applied. j, Similar to i, but for comparison of the odor identity classification accuracy. k,

Statistics of the odor identity classification accuracy for G7f and integrating both channels. Dark-colored lines represent the results of SRD, Raw, and SRD-Raw ($n = 2$, mean ± s.e.m). Each light-colored line represents a fly. One-sided Wilcoxon signed-rank test for G7f vs Both ($n = 6$). ****$P < 0.0001$, ***$P < 0.001$, **$P < 0.01$, *$P < 0.05$, ns - not significant ($P > 0.05$).

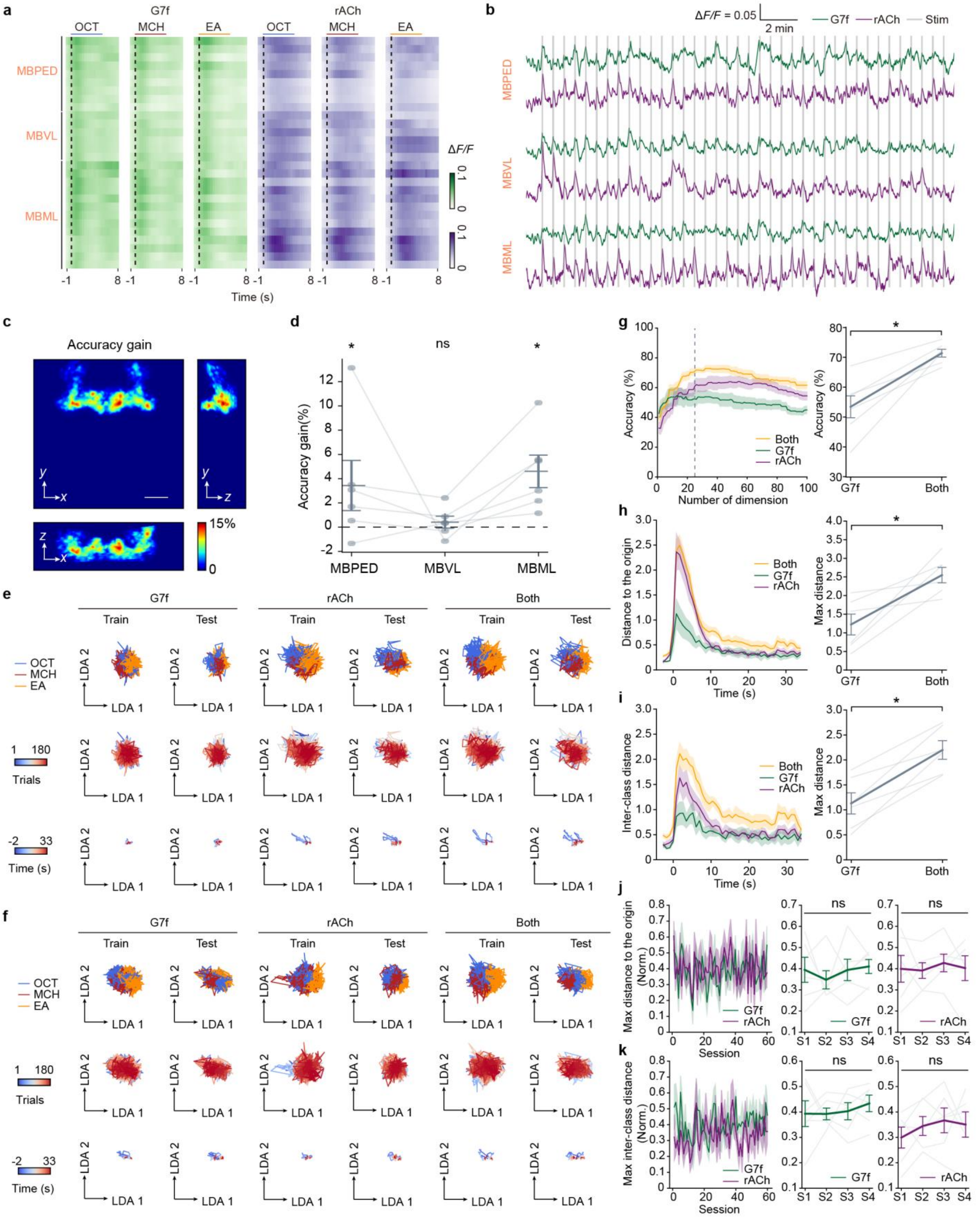

Supplementary Fig. 15.

Olfactory representation of the mushroom body cholinergic cells. a, Trial-averaged G7f and rACh responses to 3 odor stimuli of the sample voxels in the MBPED, MBVL, MBML of a fly.

Dashed lines sign the start of odor delivery. Stimulus periods are marked above (5 s; blue: OCT; red: MCH; orange: EA). b, $\Delta F/F$ traces of some voxels in the MBPED, MBVL, MBML across several trials. The gray lines sign the start of odor delivery. c, Map of decoding accuracy gain by integrating rACh dynamics, averaged across flies (n = 6). Scale bar: 50 μm. d, Average accuracy gain of MBPED, MBVL, and MBML. The dots and lines represent the values of each fly. One-sided Wilcoxon signed-rank test for each region against 0. e, f, Low-dimensional manifolds of two example flies. Top and middle: Each line represents the response in one trial. Bottom: Each line is the average odor response of an odor identity. Manifolds from 2s before odor delivery to 33s after are plotted. Top: Colors denote odor identities (blue: OCT; red: MCH; orange: EA). Middle: Colors denote the trial numbers (blue: early; red: late). Bottom: Colors denote the time relative to odor delivery (blue: early; red: late). Arrow lengths are in arbitrary units but consistent across dimensions. g, Left: The voxel-level odor identity classification accuracy changing with the dimensions of PCs retained, for G7f, rACh, and integrating both channels. Integrating both channels yields consistently improved accuracy across most tested dimensionalities. The dashed line (dimension = 25) marks the threshold taken, as the average accuracy reaches a high and stable level for all indicators. Right: Comparison of the voxel-level odor identity classification accuracy between using only the G7f channel and integrating both channels. One-sided Wilcoxon signed-rank test applied. h, i, Metrics of the manifolds for integrating both channels, G7f only and rACh only. h, Distance to the origin. Left: Distance to the origin changing with time relative to odor delivery. Right: Comparison of the maximum distance to the origin between using only the G7f channel and integrating both channels. One-sided Wilcoxon signed-rank test applied. i, Similar to h, but for Inter-class distance. j, k, Metrics of the manifold change across four stages. j, Maximum distance to the origin. k, Maximum inter-class distance. Left: Changes of the maximum distance across sessions. Right: Statistics of the maximum distance across four stages for each indicator. Two-sided Wilcoxon signed-rank test and Benjamini/Hochberg multi-comparison correction are applied. No significance is observed. Each light-colored line represents the result of a fly. n = 6, mean ± s.e.m in d, g-k. Source data are provided as a Source Data file. ****$P < 0.0001$, ***$P <$ 0.001, **$P < 0.01$, *$P < 0.05$, ns - not significant ($P > 0.05$).

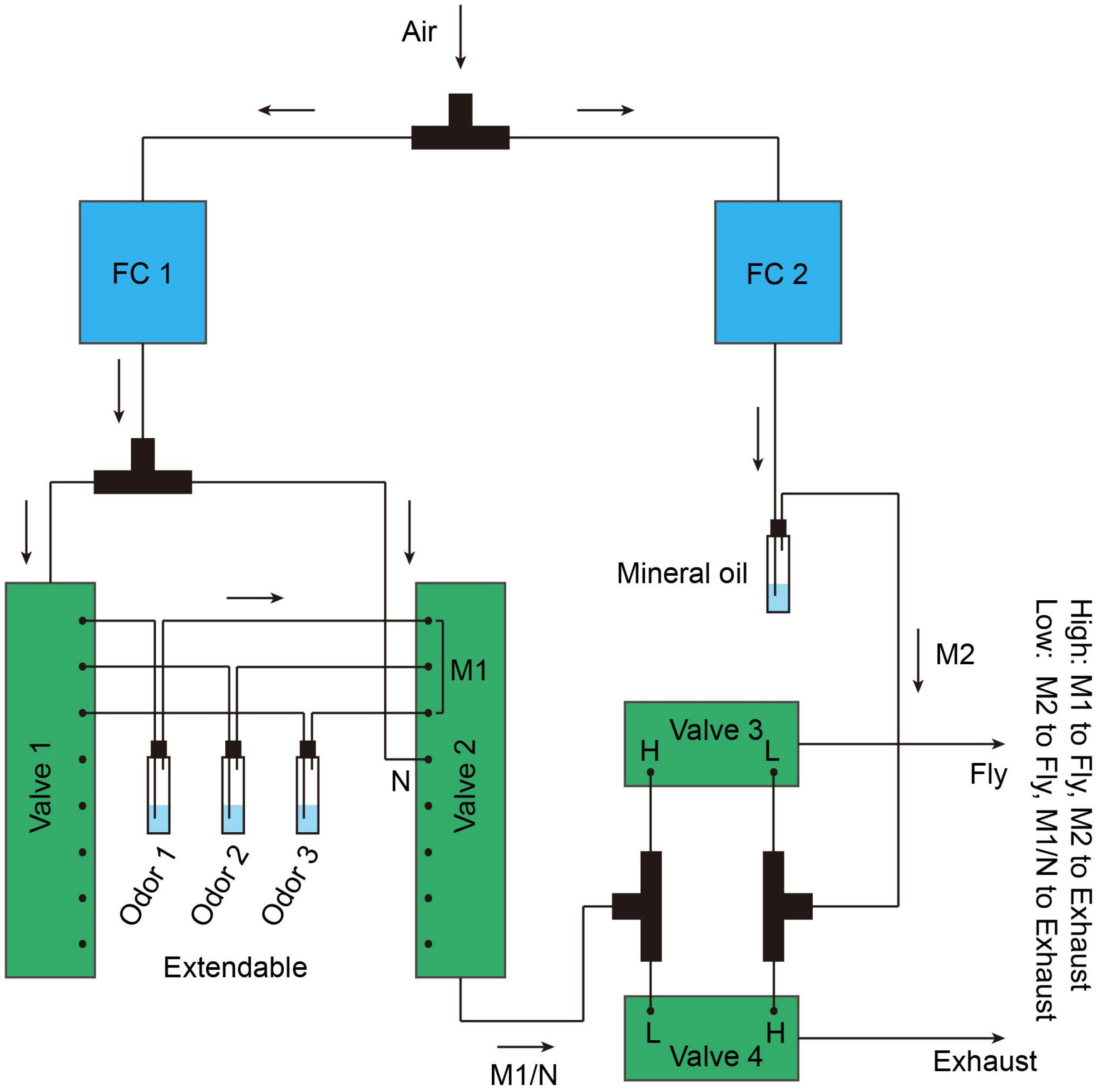

Supplementary Fig. 16.

The custom-built odor delivery system. The custom-built odor delivery system. Air is divided into two streams and fed into two fast mass flow controllers and meters, where the airflow is precisely controlled to 500 mL/min. Two combined solenoid valves are used to allow either one odor (M1, Odor 1/2/3) or air (N) to pass through. Two three-way solenoid valves are used to select the air stream delivered to the fly. Odors are generated by diluting reagents in mineral oil, while air passing through a bottle containing only mineral oil (M2) serves as the control airflow delivered to the fly during intervals. During intervals, M2 is delivered to the fly, and N is

directed to the exhaust to remove waste gas and clean the path. Before odor stimuli, the system enters a preparation state where M2 is delivered to the fly, and M1 is directed to the exhaust to prefill the path with the selected odor. During odor stimuli, M1 is delivered to the fly and M2 is directed to the exhaust. Odor number can be extended as additional ports are available on valves 1, 2. FC: fast mass flow controllers and meter. H: high potential. L: low potential.

Supplementary Table 1.

The recorded brain regions and the corresponding neuropils.

We recorded 43 brain regions in the FOV, including 20 left-side brain regions, 20 right-side brain regions, and 3 brain regions of the central complex. The numbers in the brackets after the neuropil names refer to the number of brain regions recorded. L indicates that the indices belong to the left-side brain regions, shown in Fig. 2j. Olfactory regions are labeled in coral color.

Neuropil	Brain region index	Brain region name	Full brain region name
Mushroom body (6)	64 (L)	MBPED	Pedunculus of mushroom body
	65 (L)	MBVL	Vertical lobe of mushroom body
	66 (L)	MBML	Medial lobe of mushroom body
Lateral horn (2)	55 (L)	LH	Lateral horn
Superior neuropils (6)	72 (L)	SLP	Superior lateral protocerebrum
	73 (L)	SIP	Superior intermediate protocerebrum
	74 (L)	SMP	Superior medial protocerebrum
Inferior neuropils (6)	63 (L)	CRE	Crepine
	84 (L)	SCL	Superior clamp
	59 (L)	ICL	Inferior clamp
Central and lateral complex (5)	4	NO	Nodulus
	23	EB	Ellipsoid body
	26	FB	Fan-shaped body
	56 (L)	LAL	Lateral accessory lobe
Ventrolateral neuropils (8)	79 (L)	AOTU	Anterior optic tubercle
	75 (L)	AVLP	Anterior ventrolateral protocerebrum
	76 (L)	PVLP	Posterior ventrolateral protocerebrum
	77 (L)	IVLP	Wedge
Ventromedial neuropils (8)	60 (L)	VES	Vest
	80 (L)	GOR	Gorget
	82 (L)	SPS	Superior posterior slope
	85 (L)	EPA	Epaulette
Periesophageal neuropils (2)	67 (L)	FLA	Flange
