# Supplementary material for: Prominent involvement of acetylcholine dynamics in stable olfactory representation across the Drosophila brain
Source: Nat Commun. 2025 Sep 30;16:8638. doi: 10.1038/s41467-025-63823-2 (PMC12485117; doi:10.1038/s41467-025-63823-2)
Supplement: Supplementary file 2 — Description of Additional Supplementary Files [file 41467_2025_63823_MOESM2_ESM.pdf]

Description of Additional Supplementary Files

File Name: Supplementary Movie 1
Description: Volumetric neuronal and neurochemical odor responses and low-dimensional manifolds. This video displays several trials of odor responses of two example flies. The G7f and rACh channels of the fly in Fig. 3d, e, and the r5-HT channel of the fly in Fig. 3f are shown. First row: Volumetric odor responses (mean intensity projections) of G7f, rACh, and r5-HT, from left to right. For the x-z and y-z projections, we projected 100 µm where the responses are high along the y and x directions. Second row: The experimental paradigm, process bar, time and state. The video contains trials 3 - 11 of odor stimulation. Duration from the beginning of the experiment is shown. Interval refers to the interval between odor stimulation, and OCT, MCH, EA represent the current stimulus. Third row: The manifolds of this period. The manifolds of single channels and integrating two channels are shown, as signed by the titles of the charts. In each chart, the lines are arbitrary units but indicate an equivalent length in each dimension. Scale bars: 50 µm.
