# Supplementary material for: Prominent involvement of acetylcholine dynamics in stable olfactory representation across the Drosophila brain
Source: Nat Commun. 2025 Sep 30;16:8638. doi: 10.1038/s41467-025-63823-2 (PMC12485117; doi:10.1038/s41467-025-63823-2)
Supplement: Supplementary file 4 — Reporting Summary [file 41467_2025_63823_MOESM4_ESM.pdf]

Corresponding author(s): Qionghai Dai; Lu Fang; Jiamin Wu

Last updated by author(s): Jul 25, 2025

Reporting Summary

Nature Portfolio wishes to improve the reproducibility of the work that we publish. This form provides structure for consistency and transparency in reporting. For further information on Nature Portfolio policies, see our Editorial Policies and the Editorial Policy Checklist.

Statistics

For all statistical analyses, confirm that the following items are present in the figure legend, table legend, main text, or Methods section.

n/a	Confirmed	
☐	☒	The exact sample size (*n*) for each experimental group/condition, given as a discrete number and unit of measurement
☐	☒	A statement on whether measurements were taken from distinct samples or whether the same sample was measured repeatedly
☐	☒	The statistical test(s) used AND whether they are one- or two-sided<br>*Only common tests should be described solely by name; describe more complex techniques in the Methods section.*
☐	☒	A description of all covariates tested
☐	☒	A description of any assumptions or corrections, such as tests of normality and adjustment for multiple comparisons
☐	☒	A full description of the statistical parameters including central tendency (e.g. means) or other basic estimates (e.g. regression coefficient) AND variation (e.g. standard deviation) or associated estimates of uncertainty (e.g. confidence intervals)
☐	☒	For null hypothesis testing, the test statistic (e.g. *F*, *t*, *r*) with confidence intervals, effect sizes, degrees of freedom and *P* value noted<br>*Give P values as exact values whenever suitable.*
☒	☐	For Bayesian analysis, information on the choice of priors and Markov chain Monte Carlo settings
☐	☒	For hierarchical and complex designs, identification of the appropriate level for tests and full reporting of outcomes
☐	☒	Estimates of effect sizes (e.g. Cohen's *d*, Pearson's *r*), indicating how they were calculated

Our web collection on statistics for biologists contains articles on many of the points above.

Software and code

Policy information about availability of computer code

Data collection	The data is collected by the microscope 2pSAM (Cell, 2023) and the related codes of the published version. https://mailstsinghuaeducn-my.sharepoint.com/:f:/g/personal/fjq19_mails_tsinghua_edu_cn/EtZeYbE6qfFDpNpT_uv4Mi8BiAGpYAnsJEAz9RsjXmvZdw?e=asuQ30
Data analysis	Codes for data analysis are deposited on GitHub with the link: https://github.com/jqfan77/Dual_color_fly_brain_imaging_2pSAM_analysis.

For manuscripts utilizing custom algorithms or software that are central to the research but not yet described in published literature, software must be made available to editors and reviewers. We strongly encourage code deposition in a community repository (e.g. GitHub). See the Nature Portfolio guidelines for submitting code & software for further information.

Data

Policy information about availability of data

All manuscripts must include a data availability statement. This statement should provide the following information, where applicable:

- Accession codes, unique identifiers, or web links for publicly available datasets
- A description of any restrictions on data availability
- For clinical datasets or third party data, please ensure that the statement adheres to our policy

The demo data of an example fly is available on OneDrive. We will upload the final version of the data and codes on the data platforms like Zenodo after revision. The entire dataset with a total size of 5 TB, which includes the extracted neuronal and neurochemical traces within the 3D volumes over 2h of 10 flies co-labeled by G7f and rACh and 10 flies co-labeled by G7f and r5-HT, will be open-sourced after publication, as an important resource for the neurobiology and computational neuroscience communities.

Research involving human participants, their data, or biological material

Policy information about studies with human participants or human data. See also policy information about sex, gender (identity/presentation), and sexual orientation and race, ethnicity and racism.

Reporting on sex and gender	Not relevant.
Reporting on race, ethnicity, or other socially relevant groupings	Not relevant.
Population characteristics	Not relevant.
Recruitment	Not relevant.
Ethics oversight	Not relevant.

Note that full information on the approval of the study protocol must also be provided in the manuscript.

Field-specific reporting

Please select the one below that is the best fit for your research. If you are not sure, read the appropriate sections before making your selection.

- [x] Life sciences
- [ ] Behavioural & social sciences
- [ ] Ecological, evolutionary & environmental sciences

For a reference copy of the document with all sections, see nature.com/documents/nr-reporting-summary-flat.pdf

Life sciences study design

All studies must disclose on these points even when the disclosure is negative.

Sample size	The sample size(n) of each experiment is provided in the figure/table legends in the manuscript. It is decided based on the common practice of similar studies.
Data exclusions	Data exclusions and the reasons are declared in the figure legends.
Replication	The number of repetitions for each experiment is provided in the figure/table legends in the manuscript.
Randomization	Not relevant, as there were no such experimental groups in this study.
Blinding	Not relevant, as there were no such experimental groups in this study.

Reporting for specific materials, systems and methods

We require information from authors about some types of materials, experimental systems and methods used in many studies. Here, indicate whether each material, system or method listed is relevant to your study. If you are not sure if a list item applies to your research, read the appropriate section before selecting a response.

Materials & experimental systems

n/a	Involved in the study	
☒	☐	Antibodies
☐	☒	Eukaryotic cell lines
☒	☐	Palaeontology and archaeology
☐	☒	Animals and other organisms
☒	☐	Clinical data
☒	☐	Dual use research of concern
☒	☐	Plants

Methods

n/a	Involved in the study	
☒	☐	ChIP-seq
☒	☐	Flow cytometry
☒	☐	MRI-based neuroimaging

Eukaryotic cell lines

Policy information about cell lines and Sex and Gender in Research

Cell line source(s)	HEK293T cells are used in this study to test the fluorescent indicators.
Authentication	It's a commonly used cell line and we only use them to test the indicators.
Mycoplasma contamination	All cell lines tested negative for mycoplasma contamination.
Commonly misidentified lines (See ICLAC register)	No commonly misidentified lines.

Animals and other research organisms

Policy information about studies involving animals; ARRIVE guidelines recommended for reporting animal research, and Sex and Gender in Research

Laboratory animals	2-8-day-old Drosophila melanogaster were involved in this study.
Wild animals	The study did not involve wild animals.
Reporting on sex	Female flies were selected for the brain imaging experiments considering convenience (as usually did in fly brain imaging), but sex was not considered in study design.
Field-collected samples	Flies were raised on standard cornmeal medium with a 12-h light/12-h dark cycle at 23°C and 60% humidity and housed in mixed male/female vials, and were killed under anesthesia after experiments.
Ethics oversight	No ethical approval was required for Drosophila experiments.

Note that full information on the approval of the study protocol must also be provided in the manuscript.

Plants

Seed stocks	Not relevant.
Novel plant genotypes	Not relevant.
Authentication	Not relevant.
